# The stoichiometry of the outer kinetochore is modulated by microtubule-proximal regulatory factors

**DOI:** 10.1083/jcb.201810070

**Published:** 2019-05-22

**Authors:** Karthik Dhatchinamoorthy, Jay R. Unruh, Jeffrey J. Lange, Michaella Levy, Brian D. Slaughter, Jennifer L. Gerton

**Affiliations:** 1 Stowers Institute for Medical Research, Kansas City, MO; 2 Open University, Milton Keynes, England, UK; 3 Department of Biochemistry and Molecular Biology, University of Kansas Medical Center, Kansas City, KS

## Abstract

The kinetochore is a large molecular machine that attaches chromosomes to microtubules and facilitates chromosome segregation. The kinetochore includes submodules that associate with the centromeric DNA and submodules that attach to microtubules. Additional copies of several submodules of the kinetochore are added during anaphase, including the microtubule binding module Ndc80. While the factors governing plasticity are not known, they could include regulation based on microtubule–kinetochore interactions. We report that Fin1 localizes to the microtubule-proximal edge of the kinetochore cluster during anaphase based on single-particle averaging of super-resolution images. Fin1 is required for the assembly of normal levels of Dam1 and Ndc80 submodules. Levels of Ndc80 further depend on the Dam1 microtubule binding complex. Our results suggest the stoichiometry of outer kinetochore submodules is strongly influenced by factors at the kinetochore–microtubule interface such as Fin1 and Dam1, and phosphorylation by cyclin-dependent kinase. Outer kinetochore stoichiometry is remarkably plastic and responsive to microtubule-proximal regulation.

## Abstract (precis)

Dhatchinamoorthy et al. suggest the stoichiometry of outer submodules of the budding yeast kinetochore is strongly influenced by factors at the kinetochore–microtubule interface such as Fin1 and Dam1. Outer kinetochore stoichiometry is remarkably plastic and responsive to microtubule-proximal regulation.

## Introduction

The kinetochore is a large evolutionarily conserved protein structure that is built hierarchically, from the centromeric Cse4/CENP-A nucleosome to the spindle attachments. The assembly of many of the inner components, often referred to as the constitutive centromere associated network, is necessary for the assembly of many of the outer components (De Wulf et al., 2003). The outer microtubule binding components include the Dam1 subcomplex in budding yeast, composed of 10 subunits (Li et al., 2002), which can encircle microtubules in vitro (Miranda et al., 2005; Westermann et al., 2005) and in vivo (Ng et al., 2018 *Preprint*). The microtubule-binding Ndc80 subcomplex is anchored into the kinetochore by the Mis12 or MIND complex (Dimitrova et al., 2016), both of which are evolutionarily conserved from yeast to humans. The Ndc80 complex is composed of four subunits, which form an extended coiled-coil configuration and contact the microtubule with finger-like projections (Westermann et al., 2003; Wei et al., 2005, 2007). Together these submodules, along with many additional proteins, make up the structure that attaches centromeres to microtubules for chromosome segregation (Musacchio and Desai, 2017). Elegant structural studies have elucidated many of the physical interactions and substructures that can be assembled in vitro with purified recombinant components. What these reconstitution experiments cannot reveal are the dynamics and heterogeneity that may exist in vivo.

In budding yeast, a single microtubule attaches to each chromosome for segregation at mitosis (Winey et al., 1995), making it a simple system in which to study kinetochores in vivo. Furthermore, all kinetochores cluster together to create a sub-diffraction limited spot that enables fluorescence microscopy measurements. Recent evidence based on FRAP, photoactivation, and fluorescence microscopy measurements relative to GFP suggests that the conformation of the living kinetochore in budding yeast is both plastic and cell cycle regulated, with extra copies of several submodules added in anaphase, with similar observation in fission yeast, arguing for evolutionary conservation (Dhatchinamoorthy et al., 2017). This result conflicts with previous data, and the finding is controversial and depends on the choice of reference protein (Joglekar et al., 2006). Heterogeneity in the architecture of vertebrate kinetochores has been observed and may depend on the state of microtubule attachment (Wynne and Funabiki, 2016). The mammalian Ska subcomplex is progressively loaded at congressing kinetochores to improve load-bearing capacity (Auckland et al., 2017), suggesting that proteins may load at kinetochores to improve mitotic outcome.

The two main microtubule binding components of the kinetochore in budding yeast, the Ndc80 and Dam1 submodules, display distinct behavior in anaphase. Imaging-based measurements suggest the Ndc80 subcomplex is present in ∼8 copies per kinetochore in metaphase, increasing to ∼12 in anaphase (Dhatchinamoorthy et al., 2017), with the exact number subject to debate (Joglekar et al., 2006, 2008, 2009; Haase et al., 2013). In contrast, imaging of the Dam1 subcomplex suggests it is present on kinetochores at similar levels throughout the cell cycle, with no detectable turnover (Dhatchinamoorthy et al., 2017). Consistent with the idea of a hierarchy in building the structure, submodules with strong interactions in biochemical and structural data, such as COMA, MIND, and Ndc80, display stoichiometric increases in concert during anaphase (Dhatchinamoorthy et al., 2017). The extra microtubule contacts afforded by the addition of Ndc80 submodules during anaphase may facilitate tracking along the rapidly depolymerizing microtubule. The stoichiometry of the Ndc80 submodule, but not the Dam1 submodule, was influenced by the depolymerization of microtubules, with slower depolymerization correlating with fewer copies of the Ndc80 submodule added in anaphase. The microtubule–kinetochore interface is a prime location for integrating signals to regulate kinetochore structure and function (Monda and Cheeseman, 2018). Notably, mutations affecting tension sensing and cytokinesis were examined for their impact on kinetochore stoichiometry and were found to have no effect (Dhatchinamoorthy et al., 2017). However, given the plasticity of kinetochore stoichiometry observed in vivo, regulators of the stoichiometry likely exist.

In this work we identify factors that influence outer kinetochore stoichiometry, including Fin1 (Filaments in between nuclei) and the Dam1 submodule. Fin1 was originally reported to be a spindle pole body (SPB)–related intermediate filament protein (van Hemert et al., 2002). Later, Fin1 was shown to purify with the outer kinetochore (Akiyoshi et al., 2009). Fin1 has microtubule binding activity (Woodbury and Morgan, 2007a) and stabilizes spindles (Woodbury and Morgan, 2007b). Using single-particle averaging (SPA) structured illumination microscopy (SIM), we find evidence that kinetochores are radially arranged on the spindle and demonstrate that Fin1 localizes to the SPB proximal side of kinetochore clusters. Fin1 is required for the addition of Ndc80 submodules during anaphase. Additionally, the amount of Ndc80 added in anaphase depends on another microtubule binding subcomplex, Dam1. Taken together, our data indicate that the outer kinetochore stoichiometry depends on and is responsive to cues from the microtubule–kinetochore interface.

## Results and discussion

### Fin1: A cell cycle–specific kinetochore component

**Figure 1.**
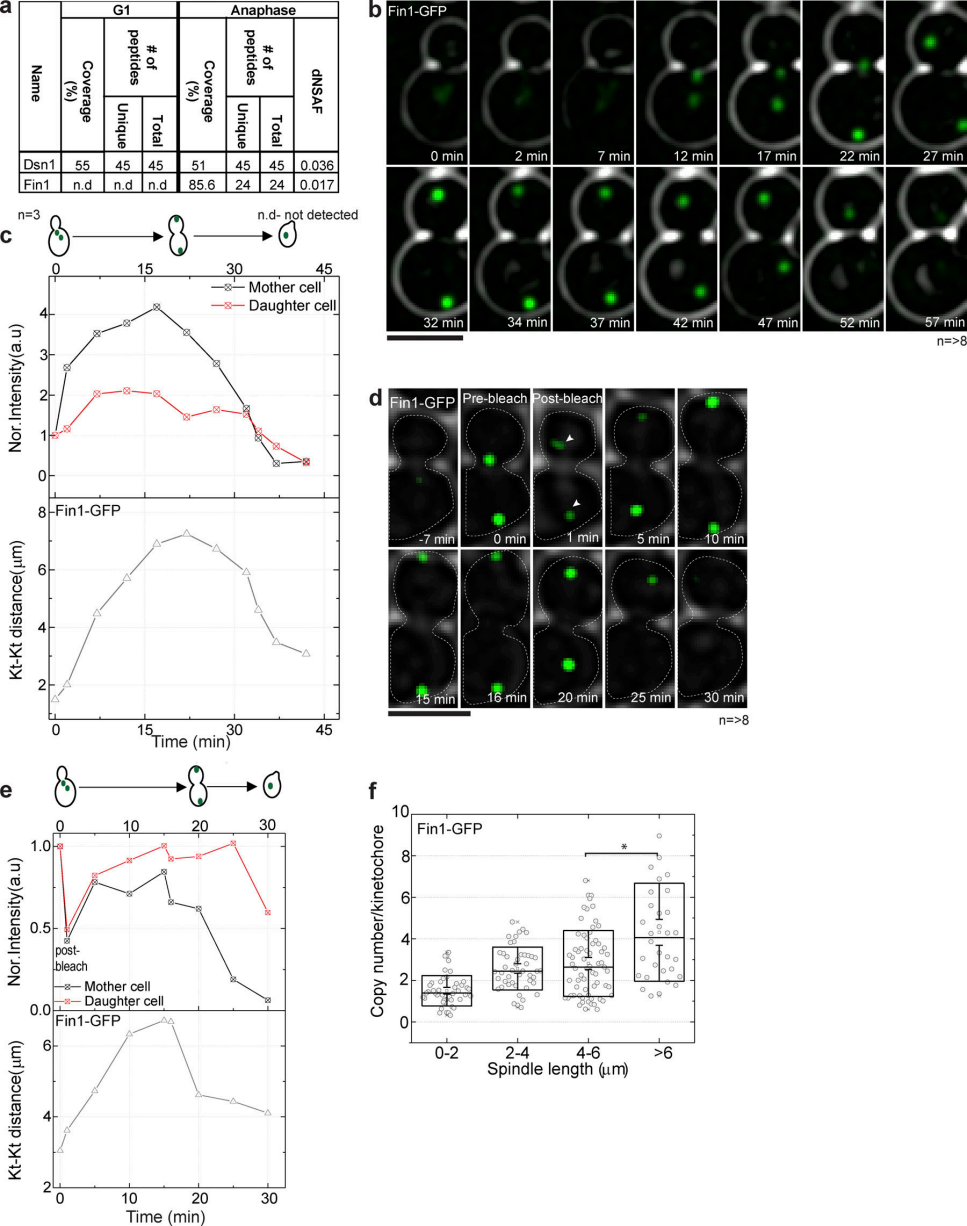
**Fin1 is associated with kinetochores from metaphase to anaphase. (a)** Dsn1-Flag was immunoprecipitated to purify kinetochore complexes from G1-arrested cultures and *cdc15-1* anaphase-arrested cultures. Purifications were performed in triplicate for each condition. MudPIT analysis of purified kinetochores detected Fin1 in the anaphase samples but not the G1 samples. **(b)** Live-cell imaging of Fin1-GFP shows that Fin1 localizes in a kinetochore-like focus from metaphase to anaphase that dissolves in G1. Eight cells were analyzed; a representative time course is shown. Bar, 5 µm. **(c)** Quantification of Fin1-GFP from b shows increased normalized intensity as the kinetochore distance increases in anaphase, and a decrease in G1. **(d)** Snapshots of FRAP on Fin1-GFP at the indicated times show recovery during anaphase following bleach in early anaphase. Heat map (lower panel) of the FRAP shows the intensity change before and after bleaching. Eight cells were analyzed; a representative time course is shown. Bar, 5 µm. **(e)** Quantification of Fin1-GFP from d shows recovery in anaphase. Intensity of mother and daughter kinetochore cluster was normalized to 1 at 0 min. The second time point represents the photobleach step. **(f)** Calibrated imaging was used to determine copy number of Fin1-GFP from metaphase to anaphase, plotted as a function of spindle length calculated from the inter-kinetochore distance. Fin1-GFP copy number increases as kinetochore clusters separate during anaphase. For the box plot, 200–250 kinetochore clusters were quantified, and for statistical analysis, a two-tailed *t* test was performed (*, P < 0.0001). In the box plot, the middle line represents the mean, the box represents SD, and whiskers represent SEM.

To discover factors that enabled the addition of extra kinetochore submodules during anaphase, we purified kinetochores from cells arrested in G1 with α factor and *cdc15-1* arrested anaphase cells using Dsn1-FLAG (Akiyoshi et al., 2009) and compared the protein composition by mass spectrometry. Mitotic exit network mutants, including *cdc15-1*, *tem1-1*, and *cdc5-1*, arrest with high levels of Ndc80 component Nuf2-GFP at a maximal interkinetochore distance (Fig. S1), suggesting that cells arrest in late anaphase with a kinetochore stoichiometry typical of late anaphase. There were only three proteins present specifically in the kinetochore purifications from anaphase (and not G1); of these three proteins, Fin1 was present at >10-fold higher levels than the other two, making it an exceptional candidate (Fig. 1 a and Table S1). Fin1 was recovered in previous kinetochore purifications, dependent on inner kinetochore protein Ndc10 (Akiyoshi et al., 2009). Fluorescence imaging of Fin1-GFP–tagged protein over the cell cycle was consistent with localization to kinetochores from metaphase to telophase (Fig. 1, b and c), mass spectrometry data (Akiyoshi et al., 2009), and other previous work (van Hemert et al., 2002).

To understand how the behavior of Fin1 compared with other kinetochore proteins in terms of dynamics during metaphase and anaphase, we used FRAP. Photobleaching during metaphase reveals that Fin1 can recover during anaphase (Fig. 1, d and e), suggesting it can load during this time window, similar to the behavior of subunits of the Mis12/MIND and Ndc80 complexes, but contrasting with subunits of the Dam1 complex, which do not recover in FRAP experiments (Dhatchinamoorthy et al., 2017). Calibrated imaging enables the calculation of the number of GFP molecules in a subdiffraction limited kinetochore cluster based on the fluorescence of GFP, as determined by fluorescence correlation spectroscopy in live cells (Shivaraju et al., 2012). Calibrated imaging is distinct from methods that have been previously used to calculate the stoichiometry of the yeast kinetochore that rely on a GFP-tagged reference protein, which may itself change with the cell cycle. Calibrated imaging of Fin1-GFP demonstrates an increase from approximately one to four per kinetochore as clusters separate, a significant gain (Fig. 1 f). Ndc80 subunits also increase in this time window, adding approximately four copies (Dhatchinamoorthy et al., 2017). Taken together, these experiments put Fin1 near kinetochores during the time window when kinetochores add copies of particular submodules and suggest the possibility that Fin1 could act as a licensing factor for this addition. Fin1 could also be considered a component of the kinetochore during this time window given its purification with outer kinetochore components (Akiyoshi et al., 2009).

### SPA-SIM reveals Fin1 is proximal to the SPB

**Figure 2.**
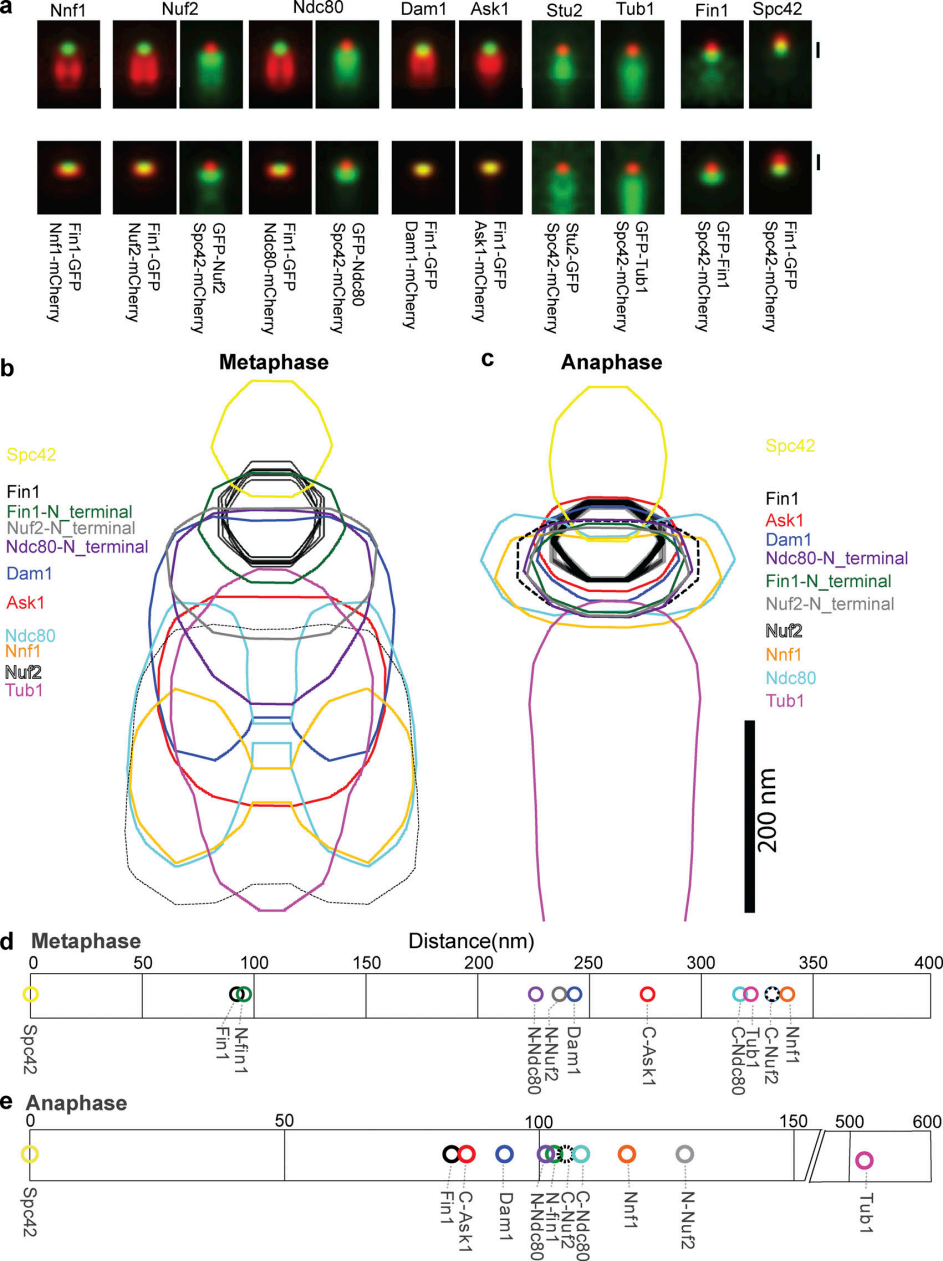
**Ultrastructural characterization of the kinetochore shows Fin1 colocalization with outer components in anaphase. (a)** SPA-SIM of kinetochore proteins in metaphase (top, SPB distance <2 µm) and anaphase (bottom, SPB distance >2 µm) aligned to either Fin1 (where present) or Spc42. The GFP-Fin1 sample is aligned to Spc42, while the Fin1-GFP sample is aligned to Fin1. Bar, 200 nm; and all images are averages of >24 individual images. **(b and c)** 75% contour maps of the data from a. **(d and e)** Distance plots for centers of Gaussian fits to the distributions in panel a.

Conventional fluorescence microscopy cannot resolve whether Fin1 colocalizes with the SPB or the kinetochore cluster. Therefore, superresolution images of GFP-tagged kinetochore proteins were collected in metaphase and anaphase to help resolve the location of Fin1 relative to kinetochore components and the SPB. SPA-SIM (Burns et al., 2015) yields a nanometer scale resolution map of GFP-tagged proteins relative to each other in the kinetochore cluster (Fig. 2). Spc42 was used as a reference for the location of the SPB, and Tub1 was used to indicate the position of the spindle microtubules. Previous experiments used point fluorescence microscopy and statistical probability maps to deduce the mean position between various proteins in the kinetochore cluster (Joglekar et al., 2009; Haase et al., 2013). In these maps, the outer kinetochore components are proximal to the SPB marker with Cse4, along with other inner kinetochore components, located more distal. The distances we calculated based on SPA-SIM measurements (Fig. 2, d and e) are consistent with these previous reports. For example, the Dam1 and Ndc80 components are SPB-proximal, while the MIND subunit Nnf1 is more distal. Furthermore, we corroborate the finding that the cluster is more elongated in metaphase but becomes compacted during anaphase (Joglekar et al., 2009).

Microtubules in mitotic spindles are organized as a cylinder (Winey et al., 1995). The organization of centromeres on the spindle in mitosis in budding yeast has been modeled, with cohesin present as a 350-nm barrel surrounding the spindle microtubules (Yeh et al., 2008). Our SPA-SIM analysis nicely revealed a bi-lobed intensity for subunits of Ndc80, and other components during metaphase. Using simulation of the data, we found that the observed distributions of these proteins, based on the parameters of our superresolution microscope, were consistent with radial location of the Ndc80 submodule in metaphase in a cylinder of ∼260 nm (Fig. S2). These results are consistent with previous models that predict the kinetochore cluster would be a barrel inside the cohesin barrel, providing experimental evidence to support the cylindrical configuration of kinetochores on the mitotic spindle in vivo.

During metaphase, Fin1 is located between the SPB and the outer components of the kinetochore. During anaphase, Fin1 more clearly colocalizes with the microtubule-binding components of the kinetochore Ndc80 and Dam1. We conclude that Fin1 is located at the outermost edge of the kinetochore cluster, at a position that would enable it to influence the outer kinetochore during metaphase and anaphase.

### Fin1 is required for normal stoichiometry of outer kinetochore submodules

**Figure 3.**
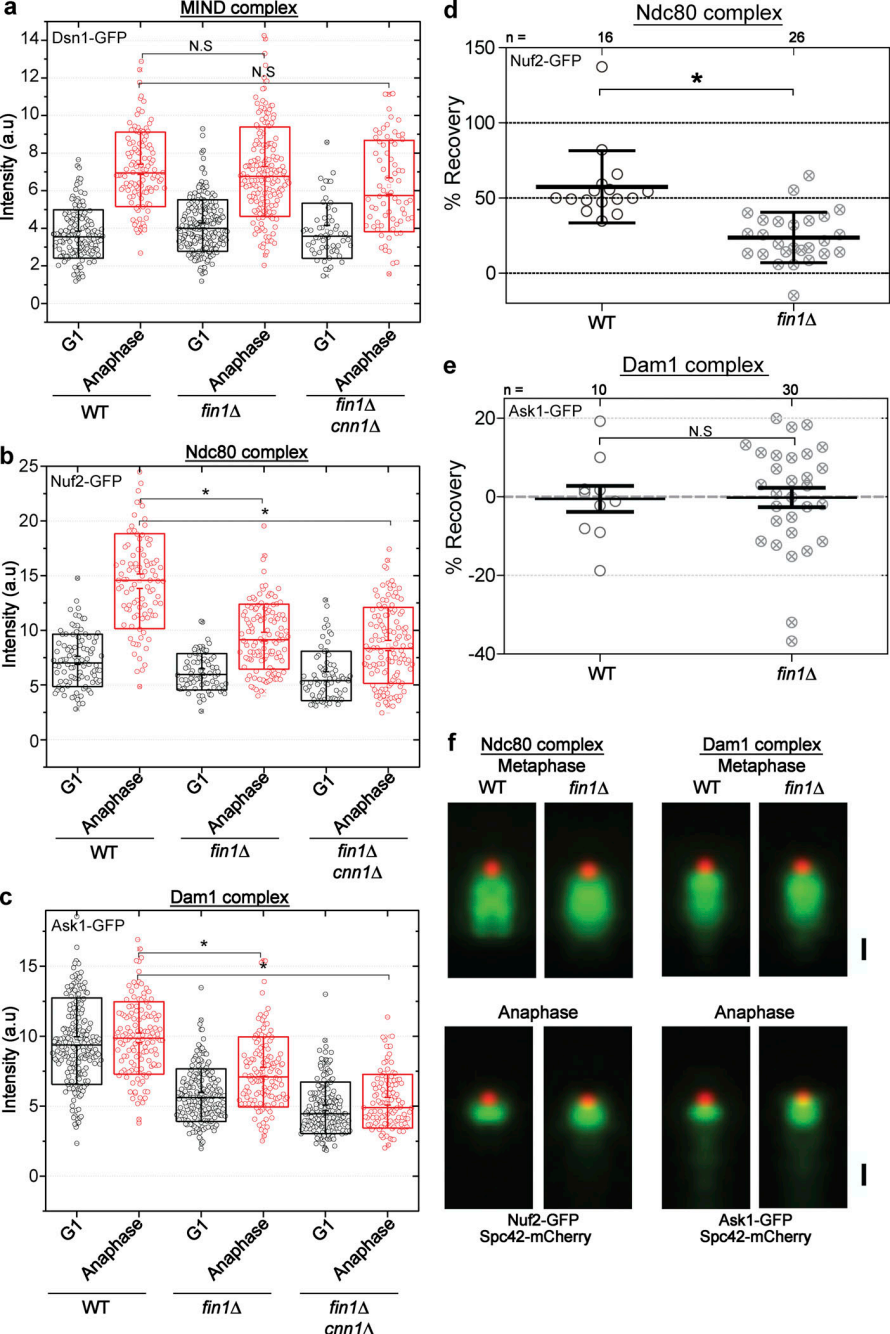
**Assembly of microtubule binding subcomplexes Dam1 and Ndc80 is affected by loss of Fin1. (a)** The MIND submodule component Dsn1 is not significantly affected by deletion of *FIN1* or *FIN1* and *CNN1.* The normalized intensity of GFP-tagged Dsn1 protein was quantified in G1 and anaphase cells from an asynchronous culture. For the box plots in a–c, 200–250 kinetochore clusters were quantified, and for statistical analysis, a two-tailed *t* test was used. The middle line represents the mean, the box represents SD, and whiskers represent SEM. **(b)** The addition of the Ndc80 submodule component Nuf2 in anaphase is significantly reduced by deletion of *FIN1* or *FIN1* and *CNN1*, while the G1 level is unchanged. The normalized intensity of GFP-tagged Nuf2 protein was quantified in G1 and anaphase cells from an asynchronous culture (*, P < 0.0001). **(c)** The level of Dam1 submodule component Ask1 does not change between G1 and anaphase in WT cells. However, lower levels overall are observed when *FIN1* or *FIN1* and *CNN1* are deleted (*, P < 0.0001). **(d)** The percent recovery of Nuf2-GFP in *fin1Δ* (*n* = 26 cells) shows significantly less recovery than in WT (*n* = 16 cells). For statistical analysis, a two-tailed *t* test was performed. In the plot, the middle line represents the mean, and whiskers represent SD; *, P, < 0.0001. **(e)** The percent recovery of Ask1-GFP is similarly poor in *fin1Δ* (*n* = 30) and WT cells (*n* = 10). Plotting and statistical analysis were performed as in d. **(f)** SPA-SIM of kinetochore clusters in metaphase (top, SPB distance <2 µm) and anaphase (bottom, SPB distance > 2 µm) aligned to Spc42 in WT and *fin1Δ* backgrounds. All images are averages of >24 individual images. Bar, 200 nm.

To examine kinetochore make-up in the absence of Fin1, we created strains without *FIN1* and GFP tags on a subunit of either the MIND, Ndc80, or Dam1 complex, as an indicator of the status of the subcomplex. We found that the normalized intensity and addition of the MIND complex was unaffected by loss of Fin1 (Fig. 3 a). The normalized intensity of Ndc80 in G1 was similarly unaffected by loss of Fin1, but the addition of Ndc80 during anaphase was strongly impaired (Fig. 3, b and c), demonstrating Fin1 is necessary for this addition. Although Dam1 does not add copies during anaphase, the overall normalized fluorescence intensity was affected by loss of Fin1, with lower levels present in G1 and anaphase (Fig. 3 c), suggesting that the assembly of Dam1 may also depend to some extent on Fin1.

To further define the function of Fin1 for the addition of Ndc80, we used FRAP (Fig. 3, d and e). We previously demonstrated that when subunits of the MIND or Ndc80 complex are photobleached during metaphase, they recover during anaphase, indicating that molecules of MIND and Ndc80 complexes are added to the kinetochore cluster as anaphase progresses, a result further confirmed using photoactivation (Dhatchinamoorthy et al., 2017). For 16 photobleached cells, the average recovery was >50%. However, the same photobleaching experiments in the *fin1Δ* background reveal a dramatically and significantly reduced recovery of fluorescence during anaphase, indicating that Fin1 facilitates the recovery (Fig. 3 d). As expected, the Dam1 complex does not recover in anaphase following photobleaching in metaphase, with or without *FIN1* (Fig. 3 e).

To further address how loss of Fin1 impacts kinetochores, we performed SPA-SIM for a subunit of the Ndc80 and Dam1 subcomplexes in a *fin1Δ* background, using Spc42-mCherry as a fiducial marker. SPA-SIM data collected for Nuf2-GFP and Ask1-GFP in the *fin1Δ* background shows that the overall organization of the kinetochore cluster is not dramatically altered (Fig. 3 f). In metaphase, the size of the Nuf2 cluster along the spindle axis is 468.2 nm (SEM, 3.9 nm) in the WT background. In the mutant background, the Nuf2 cluster is 447.7 nm (SEM, 2.1 nm). In anaphase, the Nuf2 cluster in the WT background is 194.0 nm (SEM, 1.7 nm), while the cluster in the mutant background is 255.6 nm (SEM, 2.6 nm). The Ask1 cluster in the WT background is 467.9 nm (SEM, 5.1) in metaphase, and in the mutant background, it is 440.1 nm (SEM, 3.5). In anaphase, the Ask1 cluster in the WT background is 187.2 nm (SEM, 5.0) and in the mutant background, it is 259.8 nm (SEM, 4.5). In both phases, the WT data appear slightly more clustered, though in metaphase this only appears to condense the substructures and not change the overall shape of the distribution. Although there are differences in the *fin1Δ* background compared with WT, the changes are all smaller that the difference between metaphase and anaphase, which we can effectively quantify. Furthermore, by filtering out a small subset of spots that are not diffraction-limited, we ensure that the technique is not compromised. Therefore, none of these differences are expected to compromise our ability to use fluorescence intensity measurements to quantify stoichiometric changes.

Taken together, our findings are consistent with Fin1 acting as a recruitment or licensing factor for the addition of Ndc80 in anaphase. In addition to MIND, Cnn1/CENP-T provides a critical contact for Ndc80 in the kinetochore (Schleiffer et al., 2012; Malvezzi et al., 2013), but the recruitment of additional Ndc80 in anaphase surprisingly did not require Cnn1 (Dhatchinamoorthy et al., 2017). Recent work reveals that the Cnn1/CENP-T interactions only become essential for assembly of kinetochores in vitro when the MIND pathway of recruitment is crippled (Lang et al., 2018). While Cnn1 may have the capacity to anchor extra Ndc80 into the kinetochore, loss of Cnn1 did not worsen the phenotype of the *fin1Δ* strain (Fig. 3, a–c). Importantly, the Ndc80-anchoring MIND complex is unaffected by loss of Fin1, arguing that the addition of Ndc80 in anaphase may not be automatically templated by the addition of MIND subunits, even though MIND may provide a critical anchoring interaction. In other words, the presence of the MIND complex is not sufficient for the assembly of the Ndc80 complex. The loss of Fin1 also impacts the stoichiometry of the Dam1 complex. Altogether, our results suggest the stoichiometry of the outer kinetochore may depend on Fin1-dependent spindle proximal cues.

### Interplay of Dam1 and Ndc80 on the stoichiometry of the outer kinetochore

**Figure 4.**
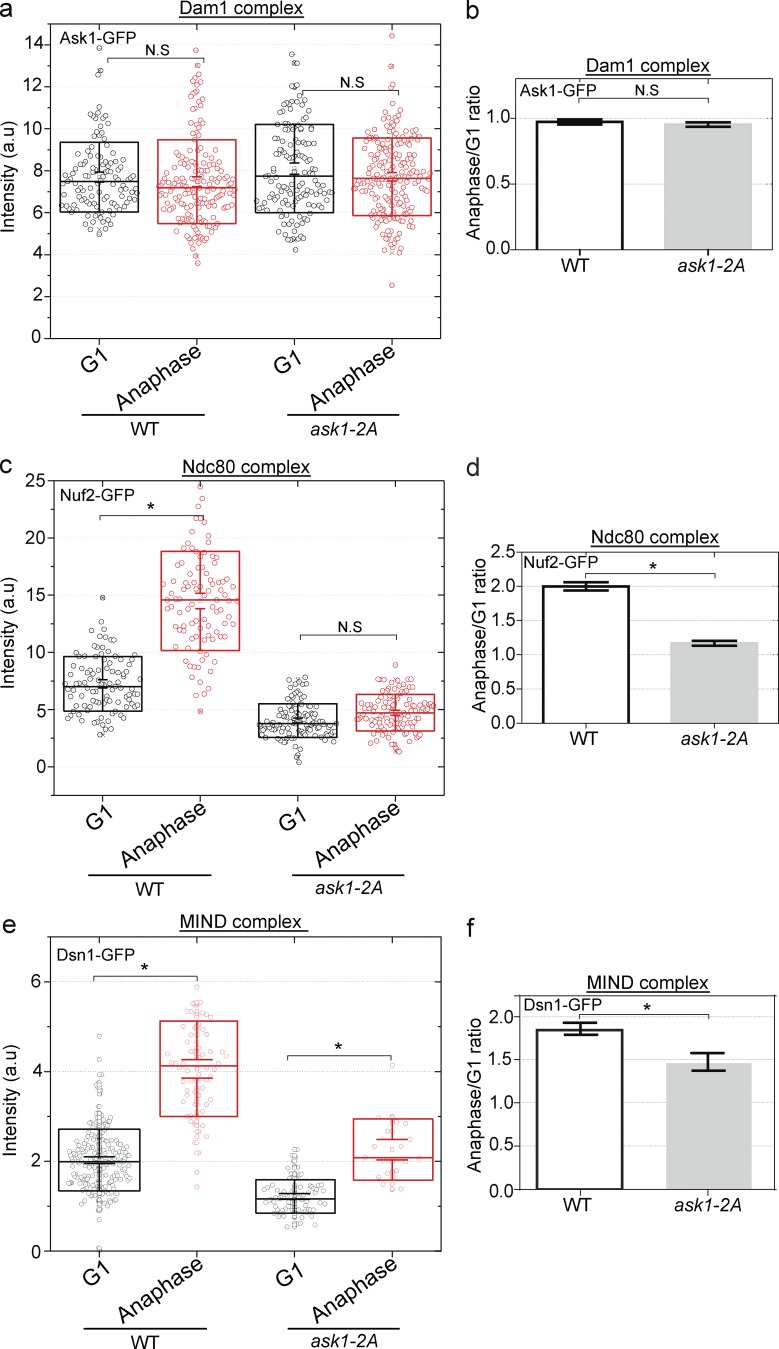
**An Ask1 phosphomutant curtails the addition of Ndc80 in anaphase. (a)** Normalized fluorescence intensity measurements indicate that a strain bearing the *ask1-2A* mutation does not compromise the assembly of the Dam1 subcomplex. For box plots (a and c), 100–150 kinetochore clusters were quantified, and for statistical analysis, a two-tailed *t* test was performed. The middle line represents the mean, the box represents SD, and whiskers represent SEM. **(b)** The ratio of fluorescence in anaphase to G1 is similar in the WT and mutant strains. Error bars indicate SEM. **(c)** Normalized fluorescence intensity measurements indicate that a strain bearing the *ask1-2A* mutation blocks the addition of Ndc80 subcomplex component Nuf2-GFP in anaphase (*, P < 0.0001). **(d)** The ratio of fluorescence in anaphase/G1 shows that Nuf2-GFP does not increase in intensity in the *ask1-2A* strain background, in contrast to the normal doubling observed in a WT strain background. Error bars indicate SEM; *, P, <0.0001. **(e)** Normalized fluorescence intensity measurements indicate that a strain bearing the *ask1-2A* mutation has addition of MIND subcomplex component Dsn1-GFP in anaphase (*, P < 0.0001). **(f)** The ratio of fluorescence in anaphase/G1 shows that Dsn1-GFP increases in intensity in the *ask1-2A* strain background. Error bars indicate SEM; *, P < 0.0001.

Dam1 and Ndc80 submodules both interact with microtubules, but also interact with each other (Shang et al., 2003; Lampert et al., 2010, 2013; Tien et al., 2010; Kim et al., 2017; Jenni and Harrison, 2018). A temperature-sensitive mutation in Dam1 impairs the addition of Ndc80 in anaphase, but the mutation also compromised the assembly of the Dam1 complex (Dhatchinamoorthy et al., 2017). Ask1 is a subunit of the Dam1 submodule. In a strain with two mutations in Cdc28/CDK phosphorylation sites in Ask1 (Higuchi and Uhlmann, 2005), Dam1 assembly is not compromised based on a normal level of fluorescence (Fig. 4). Under these conditions, the Ndc80 complex is present at lower-than-normal levels in G1, and there is no increase during anaphase, suggesting that compromising phosphorylation of the Dam1 submodule can cripple recruitment or stabilization of the Ndc80 complex. The MIND complex, however, increases in anaphase relative to G1, although not to the same degree as in the WT background, but its recruitment in anaphase is not as compromised as the Ndc80 complex by the Dam1 phosphomutation. These findings highlight the interdependence of the two microtubule-binding submodules on each other in the kinetochore clusters in living cells. These findings also emphasize some independence between the recruitment of the MIND complex and the Ndc80 complex, akin to the observations in the *fin1*Δ background. The recruitment of Ndc80 may not be solely templated by the MIND complex.

### The impact of Fin1 phosphomutants on the stoichiometry of the outer kinetochore

The kinetochore assembly state is regulated by phosphorylation of various subunits in many different species, suggesting phosphorylation acts as an evolutionarily conserved regulatory mechanism of this molecular machine (Gascoigne and Cheeseman, 2013; Wynne and Funabiki, 2015; Dimitrova et al., 2016). Fin1 phosphorylation is dependent on Clb5-Cdc28 upon synthesis in S/G2 phase, and Fin1 interacts with the Glc7p catalytic subunit of the protein phosphatase type 1 (PP1) complex when in the phosphorylated state (Mayordomo and Sanz, 2002; Loog and Morgan, 2005). However, Fin1 is dephosphorylated by Bmh1 and Bmh2 at mitosis and no longer interacts with Glc7. To further address whether Fin1 is acting in concert with Glc7 to regulate the make-up of the kinetochore, we analyzed the behavior of Nuf2-GFP in a *glc7-10* temperature-sensitive mutant background. Nuf2 showed normal addition to kinetochores during anaphase (Fig. S3). Therefore, Glc7 function is not necessary for the kinetochore transition in anaphase. We also note that Glc7 was not present in our kinetochore purifications. Therefore, the effect of Fin1 at kinetochores may be independent of Glc7.

**Figure 5.**
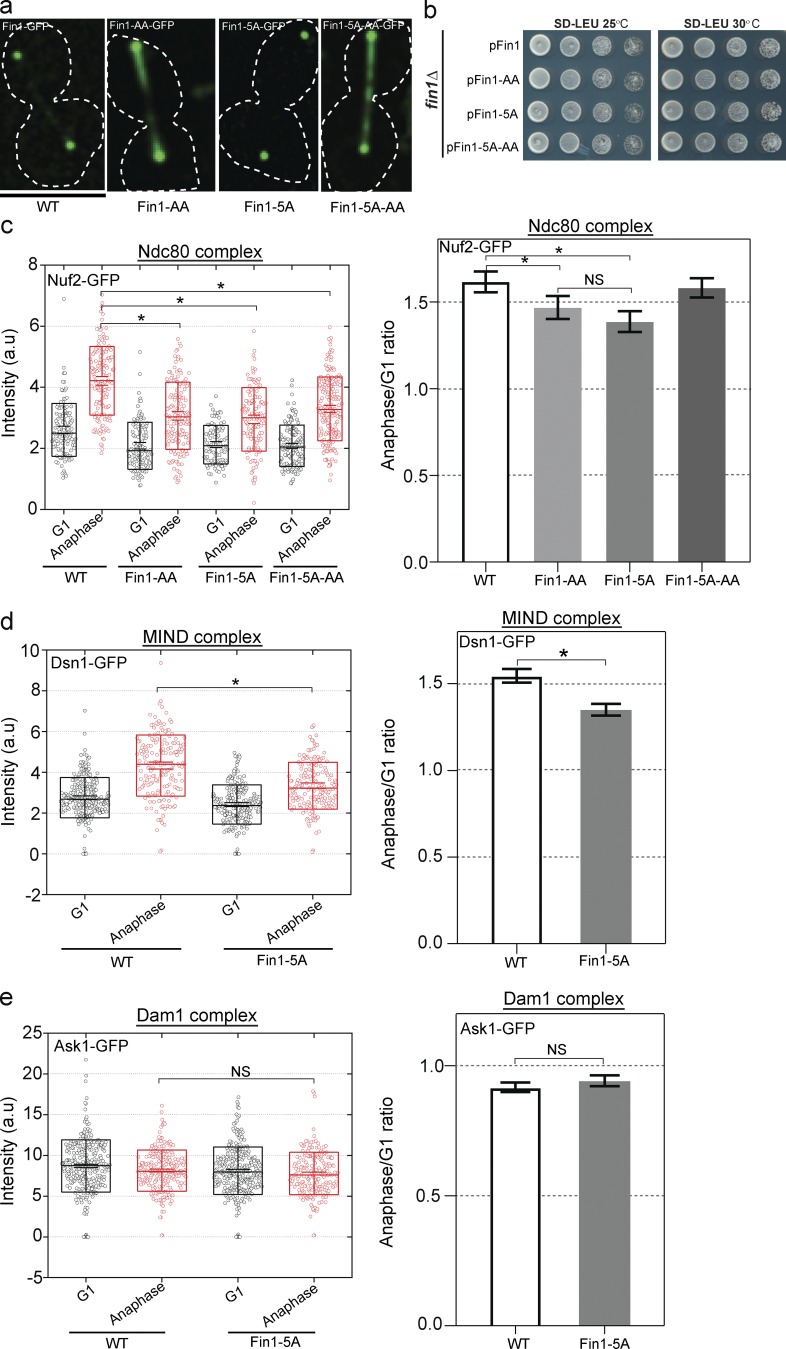
**Impact of Fin1 phosphomutants on kinetochore stoichiometry. (a)** The localization of Fin1-GFP in anaphase is shown. The only source of Fin1 is the copy on a CEN plasmid. Fin1 is expressed from its endogenous promoter. Bar, 5 µm. **(b)** The four strains in c have similar growth profiles. **(c)** The normalized fluorescence intensity measurements and anaphase/G1 ratios for Nuf2-GFP are shown. *P, <0.0001. Error bars indicate SEM. **(d)** The normalized fluorescence intensity measurements and anaphase/G1 ratios for Dsn1-GFP (MIND) in the WT and Fin1-5A background are shown. *P, <0.0001. Error bars indicate SEM. **(e)** The normalized fluorescence intensity measurements and anaphase/G1 ratios for Ask1-GFP (Dam1) in the WT and Fin1-5A background are shown. Error bars indicate SEM.

Fin1 is a phosphoprotein with 5 phosphorylation sites for cyclin-dependent kinase and a PP1 binding motif. To determine how phosphoregulation of Fin1 impacts kinetochore stoichiometry, we used point mutations in Fin1. Fin1-5A will block phosphorylation by Cdc28/CDK, whereas Fin1-AA bears a mutation in the PP1 binding motif (Bokros et al., 2016). We first examined the localization of these mutant proteins. We found that the localization of the 5A mutant protein in anaphase was indistinguishable from Fin1 (Fig. 5 a). However, when the mutant protein contained the AA mutation, it showed additional localization along the spindle, suggesting that interaction with PP1 normally prevents this localization. We next examined whether Ndc80 was added in anaphase in Fin1 phosphodeficient mutant backgrounds. Strains with these mutations as the sole source of Fin1 all grew normally under the conditions examined (Fig. 5 b). We found that Nuf2-GFP increased in anaphase relative to G1 in all the mutant backgrounds, but addition was most compromised in the Fin1-5A background (Fig. 5 c). The spindle localization of the Fin1-AA mutant did not extend to Nuf2-GFP in the AA mutant background. These data suggest phosphorylation of Fin1 may normally enhance the stoichiometry transition that depends on Fin1. Given that we observed the most significant decrease in the 5A mutant background, we next asked how Dam1 and MIND subunits were impacted by the 5A mutation. We found that the mutation also mildly reduced MIND addition (Fig. 5 d) but did not impact Dam1 (Fig. 5 e). Altogether these results suggest that interaction of Fin1 with PP1 impacts spindle localization, whereas the phosphorylation of Fin1 by Cdc28 is required for maximal addition of Ndc80 and MIND subcomplexes in anaphase. Deletion of Fin1 is more deleterious to the assembly of the Dam1 subcomplex than the phosphomutant, which has no discernable impact.

Elegant structural studies with purified proteins have revealed important principles of individual kinetochore organization, assembly, and structure. However, these studies do not reveal the dynamics, plasticity, and spindle arrangement in live cells. We provide experimental evidence that kinetochores are present in a radial arrangement on the metaphase spindle. The stoichiometry of the outer kinetochore is plastic and responsive to spindle-proximal factors, providing a possible explanation for why these proteins are variably recovered in kinetochore purification procedures from yeast extracts. Fin1, positioned at the outer edge of the kinetochore cluster from metaphase to telophase, is required to achieve the normal assembly of both Ndc80 and Dam1. The assembly of the MIND complex is not sufficient for normal assembly of the outer kinetochore in vivo, as demonstrated in Fin1 and Dam1 mutant backgrounds. The amount of Ndc80 assembled further depends on Dam1 and Fin1 phosphoregulation by cyclin-dependent kinase. While the assembly of the kinetochore is certainly hierarchical, multifactorial cues integrated at the microtubule interface can further influence the assembly status of the outer components and reveal the potential for plasticity in kinetochore stoichiometry.

## Materials and methods

Strains used in this manuscript are listed in Table S2. For statistical analysis, data distribution was judged to be normal based on visual inspection, but this was not formally tested.

### Kinetochore purification and mass spectrometry

Kinetochores were purified as described (Akiyoshi et al., 2009). To purify kinetochores from cells arrested in G1, 8 liters of culture was arrested with α-factor (5 µM). To purify kinetochores from cells arrested in anaphase, 5 liters of yeast culture was collected after shifting the culture to 37°C for 3 h. Cell pellets were frozen in liquid N_2_ and lysed using the Retsch mixer mill mm400 for three cycles (3 min on/5 min off in liquid N_2_). Lysate in H0.15 (25 mM Hepes, pH 8.0, 2 mM MgCl_2_, 0.1 mM EDTA, pH 8.0, 0.5 mM EGTA, pH 8.0, 0.1% NP-40, 150 mM KCl, 15% glycerol with 2 mM DTT, protease, and phosphatase inhibitors) was ultra-centrifuged to collect the clear top layer, which was used for affinity-purification after incubating with antibody–conjugated Dyna-beads for 3 h. Kinetochores were eluted with 0.5 mg/ml 3XFlag peptide before Multidimensional Protein Identification Technology (MudPIT).

TCA-precipitated protein samples Dsn1-His-Flag or no-tag controls isolated from three biological replicates from G1-phase or anaphase cells (12 total immunoprecipitated samples) were analyzed independently by MudPIT, as described previously (Washburn et al., 2001; Florens and Washburn, 2006). Briefly, samples were urea denatured, reduced, and alkylated before digestion with recombinant LysC (Promega) and modified trypsin (Promega). Reactions were quenched by the addition of formic acid to a final concentration of 5%. After digestion, peptide mixtures were pressure-loaded onto 100-µm fused silica microcapillary columns packed first with 9 cm of reverse-phase material (Aqua; Phenomenex), followed by 3 cm of 5-µm Strong Cation Exchange material (Luna; Phenomenex), followed by 1 cm of 5-µm C_18_ reverse-phase. The loaded microcapillary columns were placed in-line with a 1260 Quartenary HPLC (Agilent). The application of a 2.5-kV distal voltage electrosprayed the eluting peptides directly into Orbitrap-Velos Pro or Elite hybrid mass spectrometers (Thermo Fisher Scientific) equipped with a custom-made nano-liquid chromatography electrospray ionization source. Full mass spectrometry spectra were recorded on the eluting peptides over a 400–1,600 mass-to-charge range in the Orbitrap at 60,000 resolution, followed by fragmentation in the ion trap (at 35% collision energy) on the first to fifteenth most intense ions selected from the full MS spectrum. Dynamic exclusion was enabled for 90 s (Zhang et al., 2009). Mass spectrometer scan functions and HPLC solvent gradients were controlled by the XCalibur data system (Thermo Fisher Scientific).

RAW files were extracted into .ms2 file format (McDonald et al., 2004) using RawDistiller v. 1.0, in house–developed software (Zhang et al., 2011). RawDistiller D(g, 6) settings were used to abstract MS1 scan profiles by Gaussian fitting and to implement dynamic offline lock mass using six background polydimethylcyclosiloxane ions as internal calibrants (Zhang et al., 2011). MS/MS spectra were first searched using ProLuCID (Xu et al., 2015) with a peptide mass tolerance of 6 ppm and 500 ppm for fragment ions. Trypsin specificity was imposed on both ends of candidate peptides during the search against a protein database containing 5,843 yeast proteins (National Center for Biotechnology Information, February 26, 2013, release), as well as 193 usual contaminants such as human keratins, IgGs, and proteolytic enzymes. To estimate false discovery rates, each protein sequence was randomized (keeping the same amino acid composition and length), and the resulting “shuffled” sequences were added to the database, for a total search space of 12,038 amino acid sequences. Masses of 57.0215 D were differentially added to cysteine residues to account for alkylation by carboxyamidomethylcysteine and 15.9949 D were differentially added to methionine residues.

DTASelect v.1.9 (Tabb et al., 2002) was used to select and sort peptide/spectrum matches (PSMs) passing the following criteria set: PSMs were only retained if they had a DeltCn of ≥0.08; minimum XCorr values of 1.8 for singly, 2.1 for doubly, and 2.5 for triply charged spectra; peptides had to be at least seven amino acids long. Results from each sample were merged and compared using CONTRAST (Tabb et al., 2002). Combining all three runs from G1 or anaphase samples, proteins had to be detected by at least two peptides and/or two spectral counts. Proteins that were subsets of others were removed using the parsimony option in DTASelect on the proteins detected after merging all runs. Proteins that were identified by the same set of peptides (including at least one peptide unique to such protein group to distinguish between isoforms) were grouped together, and one accession number was arbitrarily considered as representative of each protein group.

*NSAF7* (Zhang et al., 2010) was used to create the final reports on all detected peptides and nonredundant proteins identified across the different runs. Spectral and protein level false discovery rates were, on average, 0.1 ± 0.09% and 2.4 ± 1.0%, respectively. *NSAF7* was also used to generate a list of all PSMs leading to the identification of proteins in either or both of the G1 and anaphase samples.

### FRAP

For FRAP and cell cycle time series experiments, an Ultraview VoX spinning-disk system (PerkinElmer) with a CSU-10 spinning disk (Yokogawa Electric Corp.) on a 200M inverted microscope base (Zeiss) was used. Images were collected with a 100× 1.46 NA α-Plan Apochromat oil objective onto an electron-multiplying charge-coupled device camera (C9100-13; Hamamatsu Photonics) using Volocity software (PerkinElmer). GFP and mCherry were excited with the 488-nm and 561-nm laser lines, respectively, using a 405/488/561/640 dichroic. The emission filter for green was a 500–550-nm Bandpass, and for red it was a 415–475-nm/580–650-nm dual Bandpass filter. For FRAP, asynchronous or arrested cells from mid-log phase were washed with synthetic complete medium and sandwiched between a slide and a coverslip in a 2% agar pad made with complete medium. Metaphase cells were chosen based on a sister kinetochore clusters distance (<0.1 µm) or SPB distance (<0.2 µm). After collecting the prebleach image, GFP was bleached with four iterations of 100% 488-nm laser. The bleached cell was followed through the next G1, and the intensity of the GFP was quantified. The intensity of the kinetochore cluster was normalized to the prebleach intensity.

For cell cycle time series experiments, the slide was prepared as described for FRAP, and images were acquired in long intervals to minimize photobleaching. For temperature-sensitive mutants, G1-arrested cells were released from the arrest and were placed on a preheated 37°C microscopy chamber for image acquisition. Similar FRAP settings were used. For quantification, ImageJ was used, and percent recovery was calculated as in Dhatchinamoorthy et al. (2017).

### Calibrated imaging (counting molecules)

Calibrated imaging to obtain the Fin1-GFP copy number was performed essentially as in Dhatchinamoorthy et al. (2017) and Shivaraju et al. (2012) with a few modifications. Fluorescence correlation spectroscopy and confocal imaging were performed on the LSM 780 microscope using a 40× 1.2 NA C-Apochromat objective. Detection was accomplished through a 488/561 dichroic and a 1–airy unit pinhole with the Quasar GaAsP spectral photomultiplier tube in photon-counting mode detecting between 500 and 561 nm. Images were acquired from an asynchronous live culture, and intensities were quantitatively compared with a cytosolic GFP strain whose concentration was determined via fluorescence correlation spectroscopy. Where necessary, the laser power was decreased to image Fin1-GFP, and images were multiplied by the power ratio to obtain appropriately scaled images for quantitation. Linearity of the microscope laser power was verified before imaging.

Image processing for calibrated imaging was performed in an identical fashion to Shivaraju et al. (2012). Briefly, the Fin1-GFP spots were fit to Gaussian functions and filtered based on size to eliminate declustered spots. Spot amplitudes were then compared with those predicted for a single GFP molecule from the fluorescence correlation spectroscopy measurements.

### Quantitative imaging (relative molecule density)

Quantitative imaging was performed similarly to the FRAP imaging (described above) with the same objective and optical path. Control and mutant samples were collected on the same day using identical settings to allow for quantitative comparison of intensity. Asynchronous live cultures were imaged with 300-nm z resolution along with transmitted light to allow for cell cycle determination. G1 cells were selected by finding clearly unbudded cells with round morphology. Anaphase cells were selected as large budded cells with kinetochores contacting either cell membrane. The maximum intensity slice for each kinetochore was fit to a Gaussian function, and kinetochores with an SD <245 nm (1.75 pixels) were included in the analysis. At this size cutoff, there is not a significant bias in the size of the G1 and anaphase kinetochore sizes for WT or mutant samples. Therefore, we are confident that reported intensity changes reflect increased protein density and not changes in kinetochore clustering. All reported intensities are Gaussian amplitudes in camera intensity units divided by 1,000 or 10,000 to put them in the 0–10 range. Anaphase/G1 ratios were calculated from the average intensities, and errors were propagated from the standard error measurements according to Bevington (2003).

### Structured illumination imaging

Structured illumination imaging was performed using an Applied Precision OMX Blaze microscope (GE Healthcare) with three PCO Edge sCMOS cameras for image detection. The objective was an Olympus 60× 1.42 NA Plan Apochromat N. GFP and mCherry were excited with 488 and 561 nm excitation, respectively, and light was collected through standard GFP and mCherry emission filters. Reconstruction was accomplished using the SoftWoRX software (GE Healthcare) following standard protocols and a Weiner filter setting of 0.001. After reconstruction, color alignment was performed using the color alignment slide and protocol provided by GE Healthcare.

### SMA analysis

SPA-SIM was performed similar to Burns et al. (2015) with custom tools in ImageJ (National Institutes of Health) with a few exceptions. Briefly, image alignment was performed with either Spc42-mCherry or Fin1-GFP as the fiducial marker for image alignment. Mother and daughter spots were identified by hand in sum projections of SIM images and then fit to two 3D Gaussian functions to identify alignment axes. Images were then realigned along this axis so that the centers of the fiducial markers were in the x and z axis center of the realigned image, and the midpoint between the fiducial markers was in the y axis center of the realigned image. The image was then divided into mother and daughter images with the fiducial centered along the y axis of the resulting image. The daughter image was flipped so that all images were oriented with fiducial markers in the center and the spindle pointing toward the bottom of the image. Early experiments did not resolve significant differences between the morphology of mother and daughter kinetochore components, so these two sets of images were averaged together in the final reconstructions.

To create SPA images, the image sets were filtered for spindle length (as described in the main text) and then maximum projected over five z slices centered on the fiducial signal. The images were then averaged and scaled eightfold with bilinear interpolation for display. Because we do not expect left-right asymmetry for any of the structures investigated here, images were averaged with their mirror images to improve signal.

For assessment of cluster positions, average vertical profiles were generated from the SPA-SIM images and fit to single Gaussian functions to estimate their centers. Despite individual image alignment, the averaged images displayed slight shifts between either Fin1 or Spc42 signals. Distances were therefore measured relative to the fiducial and corrected to the Spc42 distance with the data from Fin1-GFP and Spc42-mCherry. These distances were also used to perform fine image alignment before contour generation.

Contours were generated by scaling the images another eightfold with bilinear interpolation (for a total of 32-fold scaling over the raw data) and then thresholding at 75% of the maximum intensity in each image. An outline was then generated from this thresholded spot and added to a vector map in Adobe Illustrator.

Gaussian and multi-Gaussian fitting were performed via nonlinear least squares with error values estimated via fitting of 100 Monte Carlo simulations with random noise corresponding to the distribution of fit residuals (Bevington, 2003).

All custom plugins for this procedure are available at http://research.stowers.org/imagejplugins with source code available at https://github.com/jayunruh.

### Simulations of kinetochore cylinders

To simulate SPA-SIM imaging of a cylindrical structure, we first determined the dimensions of the microscope focal volume. We chose to use the dimensions of Spc42 from our SPA-SIM reconstructions along the direction of the spindle as determined by Gaussian fitting. The lateral dimension was determined to be 119 nm, and the axial dimension was determined to be 272 nm (full width half maximum values). These values are close to the theoretical values for mCherry imaged with a structured illumination microscope. Next, 3D images with 5 nm isotropic resolution were generated with four circles oriented along the z dimension of an image. Circles were spaced 75 nm apart along the y dimension and had a diameter of 250 nm. This image was then convolved with a 3D Gaussian function corresponding to the focal volume dimensions determined above. Finally, a single 3D Gaussian was placed on the image to approximately represent the Fin1-GFP signal.

### Online supplemental material

The three figures contain information regarding the arrest phenotype of the mitotic exit mutants with respect to Nuf2-GFP fluorescence (Fig. S1), simulations to estimate the size of the kinetochore barrel based on the parameters of our microscope (Fig. S2), and Nuf2-GFP fluorescence over the cell cycle in a *glc7-10* mutant strain background (Fig. S3). Table S1 contains a summary of the MudPIT data for kinetochore purifications, and Table S2 lists the yeast strains used.

### Original data

Original data underlying this manuscript can be accessed from the Stowers Original Data Repository at http://www.stowers.org/research/publications/libpb-1415.

## Supplementary Material

Supplemental Materials (PDF)
