# Supplementary material for: The stoichiometry of the outer kinetochore is modulated by microtubule-proximal regulatory factors
Source: J Cell Biol. 2019 May 22;218(7):2124–35. doi: 10.1083/jcb.201810070 (PMC6605801; doi:10.1083/jcb.201810070)
Supplement: Supplemental Materials (PDF) [file JCB_201810070_sm.pdf]

Supplemental material

Dhatchinamoorthy et al., https://doi.org/10.1083/jcb.201810070

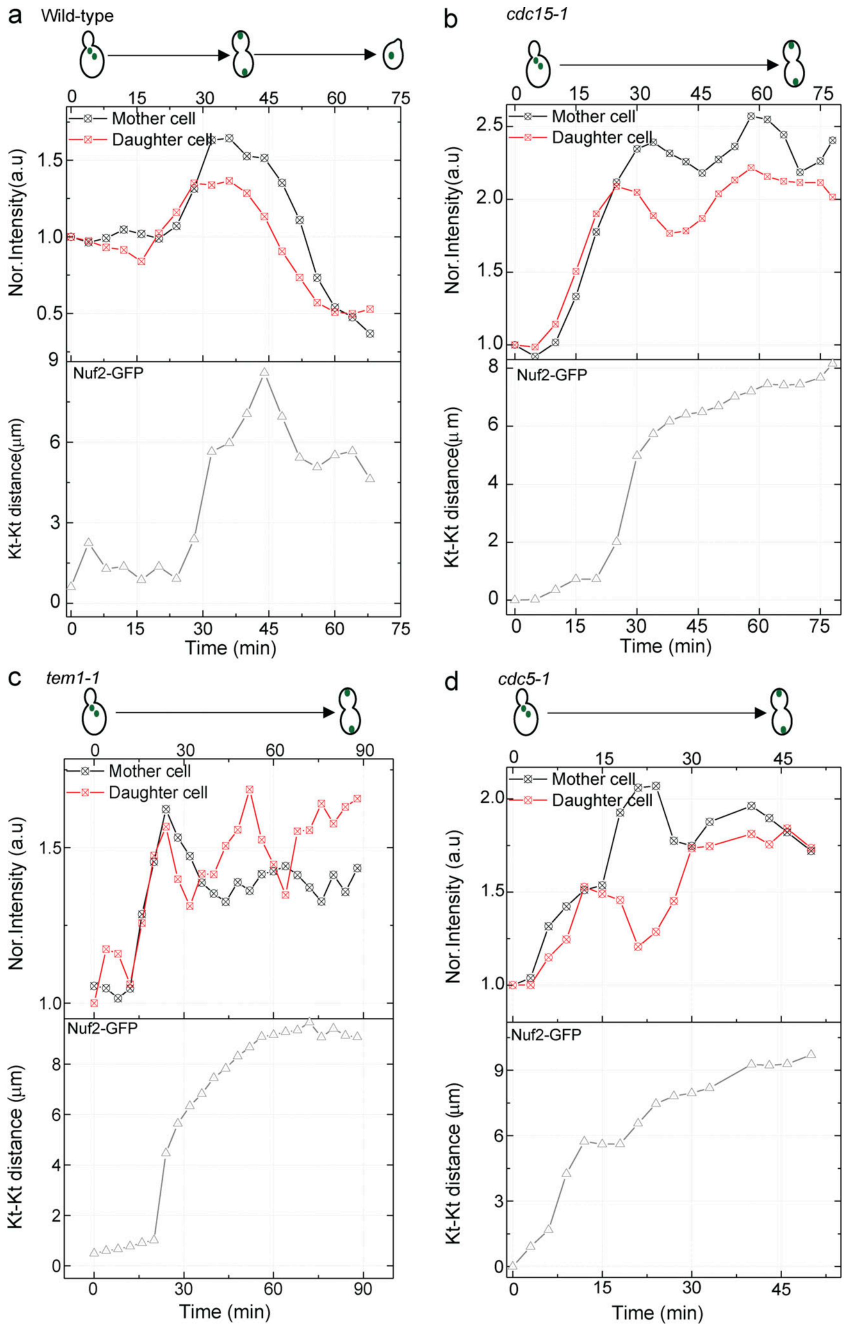

Figure S1. **Mitotic exit network mutants arrest with kinetochores in an anaphase configuration.** Live-cell imaging was performed at 37°C on a WT cell (a), a *cdc15-1* mutant cell (b), a *tem1-1* mutant cell (c), or a *cdc5-1* mutant cell (d) expressing Nuf2-GFP. The normalized fluorescence intensity of mother and daughter kinetochore clusters was quantified from metaphase to anaphase. In all three mutant backgrounds, cells arrested in late anaphase based on the interkinetochore distance, with increased fluorescence intensity. Eight cells were analyzed per genotype; a representative example is shown.

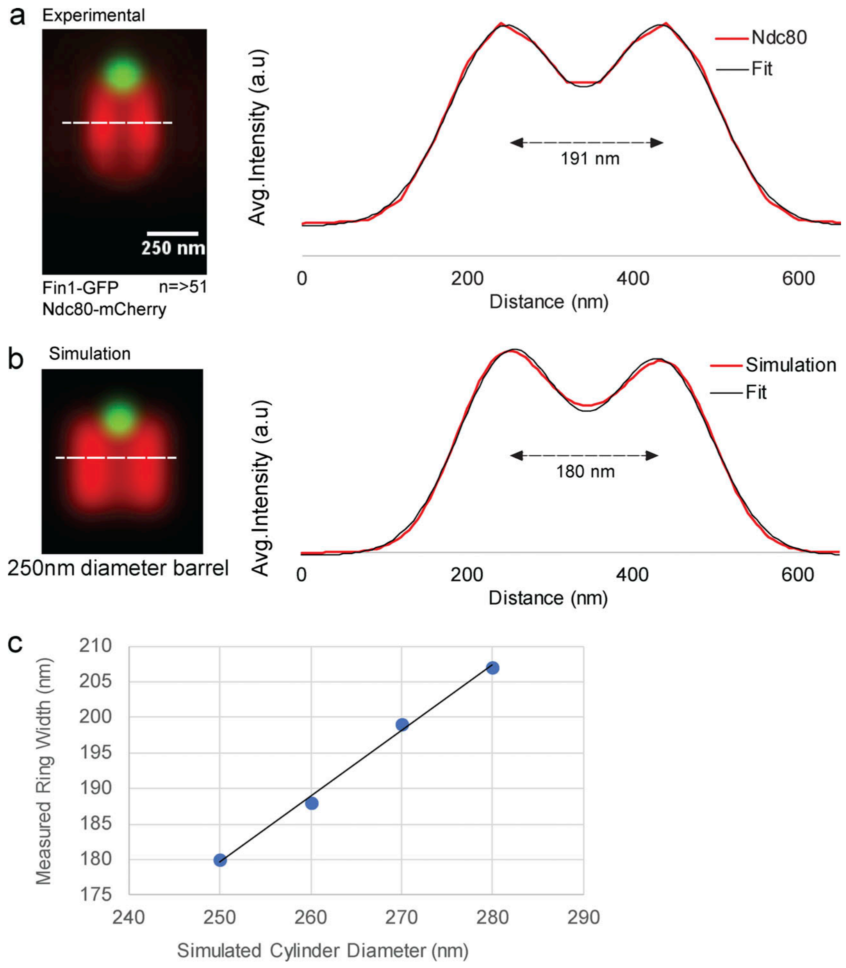

Figure S2. **Ndc80 SPA-SIM matches simulations of a cylindrical cluster. (a)** Left: Zoomed-in view of the Ndc80-GFP metaphase SPA-SIM image from Fig. 2 and profile plot position indicated by dashed line. Right: Profile plot across the kinetochore cluster and double Gaussian fit results. **(b)** Simulated SPA-SIM image of a cylinder of diameter 250 nm (red) and single point (green) as well as line profile and double Gaussian fit as in a. **(c)** Plot of measured diameter for simulated rings of different size (blue points) along with a linear trend (black).

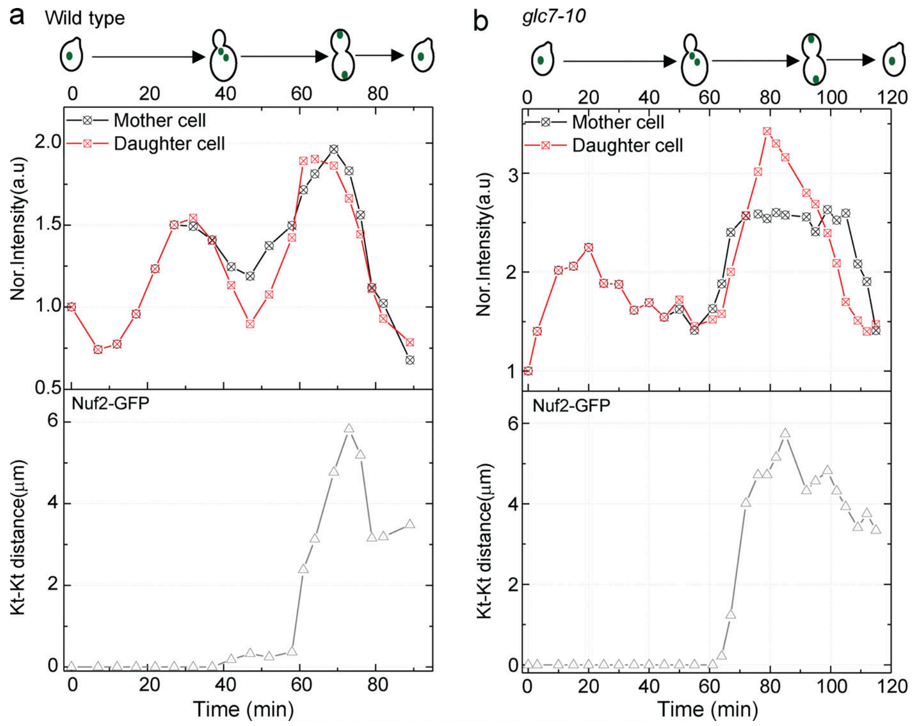

Figure S3. **Glc7 function is dispensable for the addition of Ndc80 in anaphase. (a)** α-Factor–arrested cells were released for live-cell imaging at 37°C. The normalized fluorescence intensity of Nuf2-GFP measured over the cell cycle shows the expected increase during S-phase, the expected drop as clusters split and can be detected individually, and the expected increase in anaphase followed by the expected drop in G1. **(b)** The normalized fluorescence intensity of Nuf2-GFP measured over the cell cycle shows a profile similar to WT in the *glc7-10* mutant strain background at 37°C. Eight cells were analyzed; a representative example is shown.

https://doi.org/10.1083/jcb.201810070

Table S1. **Summary of MudPIT data from kinetochore purifications**

	**G1—average (*n* = 3)**				**Anaphase—average (*n* = 3)**			
**NCBI gene**	**Peptides**	**Unique peptides**	**Sequence coverage (%)**	**dNSAF**	**Peptides**	**Unique peptides**	**Sequence coverage (%)**	**dNSAF**
CSE4	1	1	5.24	0.000118	6	6	22.56	0.002418
MIF2	8	8	19.37	0.000352	27	27	49.73	0.008705
CBF2	8	8	10.93	0.000156	5	5	6.83	0.000137
CEP3	2	2	2.30	0.000033	2	2	3.62	0.000086
SKP1	1	1	7.73	0.000135	2	2	12.89	0.000385
CTF19	2	2	6.23	0.000110	3	3	11.20	0.001247
OKP1	11	11	34.24	0.001023	19	19	51.73	0.006849
MCM21	2	2	9.42	0.000220	5	5	16.58	0.002561
AME1	9	9	30.66	0.001057	17	17	41.67	0.008692
SPC105	72	72	65.94	0.022630	71	71	58.16	0.023841
KRE28	17	17	53.77	0.019368	19	19	49.52	0.030876
MTW1	25	25	61.36	0.083183	22	22	52.60	0.065577
DSN1	45	45	55.21	0.035466	45	45	51.68	0.035596
NSL1	27	27	82.10	0.034770	22	22	69.29	0.055107
NNF1	22	22	70.64	0.067489	16	16	60.20	0.051448
NDC80	67	67	70.91	0.025597	77	77	69.61	0.050777
NUF2	33	33	59.87	0.025600	31	31	52.84	0.038770
SPC24	17	17	68.70	0.027445	18	18	63.69	0.038837
SPC25	11	11	46.15	0.014007	11	11	48.12	0.028610
DAM1	2	2	6.71	0.000034	4	4	14.87	0.000301
ASK1	n.d.	n.d.	n.d.	n.d.	4	4	20.04	0.000482
SPC19	2	2	8.48	0.000142	3	3	21.82	0.000699
SPC34	2	2	9.83	0.000080	3	3	19.67	0.000275
DUO1	2	2	10.53	0.000094	5	5	25.91	0.000904
DAD3	1	1	9.57	0.000250	1	1	9.57	0.000198
CTF3	2	2	3.41	0.000016	4	4	8.23	0.000284
CHL4	2	2	4.37	0.000063	7	7	18.12	0.000827
IML3	2	2	8.17	0.000446	4	4	21.36	0.001344
MCM22	1	1	4.18	0.000072	4	4	25.66	0.000984
CNN1	2	2	6.65	0.000049	1	1	6.28	0.000166
NKP1	4	4	21.43	0.000544	7	7	37.25	0.001164
NKP2	1	1	7.84	0.001464	1	1	13.51	0.001150
FIN1	n.d.	n.d.	n.d.	n.d.	24	24	62.20	0.017056

NCBI, National Center for Biotechnology Information; n.d., not detected.

https://doi.org/10.1083/jcb.201810070

Table S2. **Yeast strains used in this study**

Figure	Strain name	Genotype
	Budding yeast (*Saccharomyces cerevisiae*)	
Fig. 1 a	KDH49	*MATa ura3-1 leu2,3-112::pGPD1-TIR1:LEU2 his3-11 trp1-1 ade2-1 LYS2 can1-100 bar1-1 DSN1-3Flag: URA3 cdc15-2*
Fig. 1, b–d and f	KDH637	*MATa his3Δ1 leu2Δ0 met15Δ0 ura3Δ0 Fin1-GFP::URA3*
Fig. 2 a	KDH362	*MATa his3Δ1 leu2Δ0 met15Δ0 ura3Δ0 Dm1-GFP::URA3*
Fig. 3, b, d, and e; Fig. 4, a and b; Fig. S1 a; Fig. S3 a	KDH180.1	*MATa his3Δ1 leu2Δ0 met15Δ0 ura3Δ0 Nuf2-GFP::URA3*
Fig. 3 c; Fig. 4, c and d	KDH233	*MATa his3Δ1 leu2Δ0 met15Δ0 ura3Δ0 Ask1-GFP::HIS3*
Fig. 2, a–c	KDH926	*MATa his3Δ1 leu2Δ0 met15Δ0 ura3Δ0 Fin1-GFP::URA3,Nnf1-mCherry::HIS3*
	KDH929	*MATa his3Δ1 leu2Δ0 met15Δ0 ura3Δ0 Fin1-GFP::URA3,Nuf2-mCherry::HIS3*
	KDH930	*MATa his3Δ1 leu2Δ0 met15Δ0 ura3Δ0 Fin1-GFP::URA3,Ndc80-mCherry::HIS3*
	KDH931	*MATa his3Δ1 leu2Δ0 met15Δ0 ura3Δ0 Fin1-GFP::URA3,Ask1-mCherry::HIS3*
	KDH932	*MATa his3Δ1 leu2Δ0 met15Δ0 ura3Δ0 Fin1-GFP::URA3,Dam1-mCherry::HIS3*
	KDH960	*MATa his3Δ1 leu2Δ0 met15Δ0 ura3Δ0 Fin1-GFP::URA3,pSpc42-mCherry::LEU2*
	KDH1035	*MATa his3Δ1 leu2Δ0 met15Δ0 ura3Δ0 GFP-Nuf2,pSpc42-mCherry::LEU2*
	KDH1036	*MATa his3Δ1 leu2Δ0 met15Δ0 ura3Δ0 GFP-Ndc80,pSpc42-mCherry::LEU2*
	KDH946	*MATa his3Δ1 leu2Δ0 met15Δ0 ura3Δ0 GFP-Tub1::KAN,pSpc42-mCherry::LEU2*
	KDH950	*MATa his3Δ1 leu2Δ0 met15Δ0 ura3Δ0 Stu2-GFP::URA3,pSpc42-mCherry::LEU2*
Fig. 3 a	KDH915	*MATa his3Δ1 leu2Δ0 met15Δ0 ura3Δ0 Dsn1-GFP::URA3, fin1Δ:: KANMX6*
	KDH924	*MATa his3Δ1 leu2Δ0 met15Δ0 ura3Δ0 Dsn1-GFP::URA3, fin1Δ:: KANMX6, cnn1Δ:: NATMX6*
Fig. 3 b	KDH923	*MATa his3Δ1 leu2Δ0 met15Δ0 ura3Δ0 Nuf2-GFP::URA3,fin1Δ:: KANMX6*
	KDH925	*MATa his3Δ1 leu2Δ0 met15Δ0 ura3Δ0 Nuf2-GFP::URA3, fin1Δ:: KANMX6,cnn1Δ:: NATMX6*
Fig. 3 c	KDH831	*MATa his3Δ1 leu2Δ0 met15Δ0 ura3Δ0 Ask1-GFP::URA3,fin1Δ:: KANMX6*
	KDH902	*MATa his3Δ1 leu2Δ0 met15Δ0 ura3Δ0 Ask1-GFP::URA3,fin1Δ:: KANMX6, cnn1Δ:: NATMX6*
Fig. 3 f	KDH38	*MATa his3Δ1 leu2Δ0 met15Δ0 ura3Δ0 Nuf2-GFP::HIS,Spc42-mCherry::LEU2*
	KDH41	*MATa his3Δ1 leu2Δ0 met15Δ0 ura3Δ0 Ask1-GFP::HIS,Spc42-mCherry::LEU2*
	KDH942	*MATa his3Δ1 leu2Δ0 met15Δ0 ura3Δ0 Ask1-GFP::HIS,Spc42-mCherry::LEU2,fin1Δ::KANMX6*
	KDH953	*MATa his3Δ1 leu2Δ0 met15Δ0 ura3Δ0* Nuf2-GFP::HIS,Spc42-mCherry::LEU2,*fin1Δ::KANMX6*
Fig. 4, a and b	KDH1000	*MATa cdc14-1,leu2-3,ura3,trp1-1,his3-11,15,can1-100,GAL,psi+,ASK-S216A,S250A-Myc9:: LEU2,cdc5L158G,Nuf2-GFP::URA3*
Fig. 4, c and d	KDH987	*MATa cdc14-1,leu2-3,ura3,trp1-1,his3-11,15,can1-100,GAL,psi+,ASK-S216A,S250A-GFP:: URA3,cdc5L158G*
Fig. 4, e and f	MM593	*MATa cdc14-1,leu2-3,ura3,trp1-1,his3-11,15,can1-100,GAL,psi+,ASK-S216A,S250A-Myc9:: LEU2,cdc5L158G DSN1-GFP::URA*
	KDH179	*MATa his3Δ1 leu2Δ0 met15Δ0 ura3Δ0 DSN1-GFP::URA3*
Fig. 5 a	KDH845	*MATa his3Δ1 leu2Δ0 met15Δ0 ura3Δ0 fin1Δ:: KANMX6 pFIN1-GFP::LEU2*
	KDH846	*MATa his3Δ1 leu2Δ0 met15Δ0 ura3Δ0 fin1Δ:: KANMX6 pfin1-AA-GFP::LEU2*
	KDH847	*MATa his3Δ1 leu2Δ0 met15Δ0 ura3Δ0 fin1Δ:: KANMX6 pfin1-5A-GFP::LEU2*
	KDH848	*MATa his3Δ1 leu2Δ0 met15Δ0 ura3Δ0 fin1Δ:: KANMX6 pfin1-5A-AA-GFP::LEU2*
Fig. 5, b and c	MM594	*MATa his3Δ1 leu2Δ0 met15Δ0 ura3Δ0 Nuf2-GFP::URA3,fin1Δ:: KANMX6 pFIN1::LEU2*
	MM595	*MATa his3Δ1 leu2Δ0 met15Δ0 ura3Δ0 Nuf2-GFP::URA3,fin1Δ:: KANMX6 pfin1-AA::LEU2*
	MM596	*MATa his3Δ1 leu2Δ0 met15Δ0 ura3Δ0 Nuf2-GFP::URA3,fin1Δ:: KANMX6 pfin1-5A::LEU2*
	MM597	*MATa his3Δ1 leu2Δ0 met15Δ0 ura3Δ0 Nuf2-GFP::URA3,fin1Δ:: KANMX6 pfin1-5A-AA::LEU2*
Fig. 5 d	MM600	*MATa his3Δ1 leu2Δ0 met15Δ0 ura3Δ0 Dsn1-GFP::URA3, fin1Δ:: KANMX6 pFIN1::LEU2*
	MM601	*MATa his3Δ1 leu2Δ0 met15Δ0 ura3Δ0 Dsn1-GFP::URA3, fin1Δ:: KANMX6 pfin1-5A::LEU2*

Table S2. **Yeast strains used in this study (*Continued*)**

Figure	Strain name	Genotype
Fig. 5 e	MM598	*MATa his3Δ1 leu2Δ0 met15Δ0 ura3Δ0 Ask1-GFP::URA3,fin1Δ:: KANMX6 pFIN1::LEU2*
	MM599	*MATa his3Δ1 leu2Δ0 met15Δ0 ura3Δ0 Ask1-GFP::URA3,fin1Δ:: KANMX6 pfin1-5A::LEU2*
Fig. 1 a; Fig. S1 b	KDH383	*MATa his3Δ1 leu2Δ0 met15Δ0 ura3Δ0 Nuf2-GFP::URA3,cdc15-1:: KANMX6*
Fig. S1 c	KDH477	*MATa his3Δ1 leu2Δ0 met15Δ0 ura3Δ0 Nuf2-GFP::URA3,tem1-1:: KANMX6*
Fig. S1 d	KDH486	*MATa his3Δ1 leu2Δ0 met15Δ0 ura3Δ0 Nuf2-GFP::URA3,cdc5-1:: KANMX6*
Fig. S3 b	KDH381	*MATa his3Δ1 leu2Δ0 met15Δ0 ura3Δ0 Nuf2-GFP::URA3,glc7-1:: KANMX6*
